# Increased circulating IgG levels, myocardial immune cells and IgG deposits support a role for an immune response in pre‐ and end‐stage heart failure

**DOI:** 10.1111/jcmm.14619

**Published:** 2019-09-26

**Authors:** Patricia van den Hoogen, Saskia C. A. de Jager, Manon M. H. Huibers, Arjan H. Schoneveld, Yustina M. Puspitasari, Gideon B. Valstar, Marish I. F. J. Oerlemans, Roel A. de Weger, Pieter A. Doevendans, Hester M. den Ruijter, Jon D. Laman, Aryan Vink, Joost P. G. Sluijter

**Affiliations:** ^1^ Laboratory of Experimental Cardiology, UMC Utrecht Regenerative Medicine Center University Medical Center Utrecht Utrecht The Netherlands; ^2^ Department of Pathology University Medical Center Utrecht Utrecht The Netherlands; ^3^ Department of Genetics University Medical Center Utrecht Utrecht The Netherlands; ^4^ Laboratory of Clinical Chemistry & Haematology, ARCADIA University Medical Center Utrecht Utrecht The Netherlands; ^5^ Center for Molecular Cardiology University of Zurich Zurich Switzerland; ^6^ Department of Cardiology University Medical Center Utrecht Utrecht The Netherlands; ^7^ Heart and Lungs, Experimental Cardiology Netherlands Heart Institute (NHI) Utrecht The Netherlands; ^8^ Centraal Militair Hospitaal (CMH) Utrecht The Netherlands; ^9^ Department of Biomedical Sciences of Cells and Systems (BSCS) University Medical Center Groningen Groningen The Netherlands

**Keywords:** autoantibodies, autoimmunity, B cells, biomarker, cardiomyopathy, inflammation

## Abstract

The chronic inflammatory response plays an important role in adverse cardiac remodelling and the development of heart failure (HF). There is also evidence that in the pathogenesis of several cardiovascular diseases, chronic inflammation is accompanied by antibody and complement deposits in the heart, suggestive of a true autoimmune response. However, the role of antibody‐mediated immune responses in HF progression is less clear. We assessed whether immune cell infiltration and immunoglobulin levels are associated with HF type and disease stage, taking sex differences into account. We found IgG deposits and increased infiltration of immune cells in the affected myocardium of patients with end‐stage HF with reduced ejection fraction (HFrEF, n = 20). Circulating levels of IgG1 and IgG3 were elevated in these patients. Furthermore, the percentage of transitional/regulatory B cells was decreased (from 6.9% to 2.4%) compared with healthy controls (n = 5). Similarly, increased levels of circulating IgG1 and IgG3 were observed in men with left ventricular diastolic dysfunction (LVDD, n = 5), possibly an early stage of HF with preserved EF (HFpEF). In conclusion, IgG deposits and infiltrates of immune cells are present in end‐stage HFrEF. In addition, both LVDD patients and end‐stage HFrEF patients show elevated levels of circulating IgG1 and IgG3, suggesting an antibody‐mediated immune response upon cardiac remodelling, which in the early phase of remodelling appear to differ between men and women. These immunoglobulin subclasses might be used as marker for pre‐stage HF and its progression. Future identification of auto‐antigens might open possibilities for new therapeutic interventions.

## 1 INTRODUCTION

Heart failure (HF) is a clinical syndrome that affects approximately 1%‐2% of people in the western world.^1^ HF is caused by structural or functional cardiac abnormalities, resulting in a reduced cardiac output or increased filling pressures.^2^ This can be caused by systolic dysfunction leading to HF with reduced ejection fraction (HFrEF), or by diastolic dysfunction leading to HF with preserved EF (HFpEF).^2^ A large proportion of patients with HFrEF are diagnosed with ischaemic heart disease (IHD), because of the consequences of an acute myocardial infarction.^3^, ^4^ Another cause of systolic HF is dilated cardiomyopathy (DCM), in which the heart is functionally decompensating as a result of genetic pathogenic mutations or other causes, such as viral myocarditis.^2^, ^5^, ^6^

Regardless of HF aetiology, cardiac remodelling is one of the hallmarks, in which HF progressively worsens by adverse remodelling of the heart to compensate for losses in contractility or impaired relaxation with increased filling pressures.^7^, ^8^ One of the main players in adverse cardiac remodelling is the inflammatory response.^8^, ^9^, ^10^, ^11^ In the acute phase upon a myocardial infarction, mainly neutrophils and monocytes are important for the clearance of necrotic cells and debris.^10^ The chronic phase of remodelling is hallmarked by a prolonged inflammatory response. It has been suggested that this chronic inflammatory response has detrimental effects on the heart because of chronic activation of macrophages and B and T lymphocytes.^11^ These leucocytes secrete pro‐inflammatory cytokines, growth factors and immunoglobulins, thereby inducing adverse cardiac remodelling.^11^, ^12^, ^13^ Clinical trials targeting the chronic pro‐inflammatory response by immuno‐adsorption showed beneficial effects on mortality in patients with DCM as clearance of all four immunoglobulin G (IgG) subclasses improved cardiac performance.^14^, ^15^, ^16^, ^17^, ^18^ Conversely, general inhibition of the immune response by corticosteroids or intravenous immunoglobulin (IVIG) administration showed no effect on mortality in HF patients compared with conventional treatment.^19^, ^20^, ^21^, ^22^, ^23^ These previous observations suggest a pathogenic role of a specific antibody‐mediated immune response. In line with this hypothesis, deposits of antibodies (mainly IgG1 and IgG3) in the myocardium of HF patients have been observed. These antibodies, reacting to cardiac tissue, are bound to the failing myocardium where they induce complement activation and most likely play an important role in HF progression.^24^, ^25^, ^26^, ^27^, ^28^, ^29^ In previous studies, circumstantial evidence has been provided for an antibody‐mediated immune response in end‐stage HF. However, the question remains whether this antibody‐mediated immune response is associated with the severity of HF. In addition, it remains to be elucidated whether this immune response is a generalized phenomenon, or that differences between sex and HF aetiology exist.

Therefore, we investigated the presence of a potential antibody‐mediated immune response in patients with end‐stage HFrEF, including IHD and DCM. We investigated the presence and localization of different immunoglobulin subclasses and immune cells locally in the myocardium as well as in the circulation. In order to establish whether immunoglobulins are also detectable in the earliest phase of HFpEF, we measured immunoglobulin levels in patients with different stages of left ventricular diastolic dysfunction (LVDD).

## 2 MATERIAL AND METHODS

### 2.1 Patient population with end‐stage heart failure

Patient's myocardial tissue was stored in the cardiac tissue biobank of the University Medical Centre Utrecht in compliance with the *Declaration of Helsinki*.^30^ The study was approved by the local medical ethics committee (METC, reference number 12/387). Written informed consent for collection and biobanking of tissue samples and blood was obtained prior to transplantation or, in certain cases, approved by the ethics committee when obtaining informed consent was not possible because of death of the patient. Myocardial tissue from 10 IHD patients and 10 DCM patients was obtained from the explanted heart during heart transplantation (HTx; Table S1). Patients carrying a left ventricular assist device (LVAD) prior to transplantation were not included. Three control hearts, two donor hearts not used for transplantation and one heart obtained at autopsy, were used as reference. Fresh plasma samples of age‐ and sex‐matched IHD (n = 9) and DCM (n = 7) patients prior to HTx (2018‐2019) were collected and compared to fresh plasma samples of healthy controls without cardiovascular disease history (n = 21).

### 2.2 Patient population with early left ventricular diastolic dysfunction

Plasma of 260 patients with different stages of diastolic dysfunction was collected in the HELPFul study. HELPFul is an ongoing single centre, prospective observational study conducted at a cardiac diagnostic outpatient centre in the Netherlands^31^ (Table S2). Eligible patients were persons aged 45 years or older referred by their general practitioner for evaluation of a cardiac cause of symptoms, for example chest discomfort, shortness of breath and palpitations. Patients who had a history of coronary intervention, cardiac (bypass) surgery or with congenital heart disease were excluded. Written informed consent was obtained from all participants. The ethics committee approved the study (reference number NTR6016). Patients were categorized using the diagnostic algorithm presented at the ESC congress in Munich by the Heart Failure Association (2018). The algorithm is a scoring system to estimate the likelihood of HFpEF, which ranges from zero to six points based on minor or major abnormalities of echocardiographic diastolic function parameters (ie septal and lateral early diastolic mitral annular recoil velocity [e′], ratio of peak early [E] diastolic filling velocity to average e′ [E/e′ ratio], left atrial volume index [LAVI], left ventricular mass index [LVMI], tricuspid regurgitation velocity, relative wall thickness [RWT] and left ventricular wall thickness) and levels of natriuretic peptides. The algorithm is proposed to categorize patients into three groups: no HFpEF (HFpEF likelihood score 0‐1), indeterminate for HFpEF (HFpEF likelihood score 2‐4) and definite HFpEF (HFpEF likelihood score 5‐6). However, as the algorithm score uses diastolic function criteria and natriuretic peptides, we interpreted it as a diastolic function score with no LVDD, indeterminate LVDD and definite LVDD, respectively.

### 2.3 Myocardial tissue selection

Myocardial tissues were transversally sliced, thereby obtaining cross‐sectional overviews of the diseased heart. Non‐ischaemic regions of a mid‐ventricular heart slice of the left ventricle were selected using haematoxylin and eosin (H&E)‐ and Masson trichrome‐stained sections using light microscopy. These remote sections were defined as regions with little fibrosis and the absence of necrotic tissue. The epicardial layer and any adipose tissue, if present, were removed from the tissue in further analyses. Both paraffin‐embedded and cryosections were obtained from the myocardium.

### 2.4 Immunohistochemistry (IHC) for the detection of inflammatory cells

Tissue sections (4 μm) of formalin‐fixed and paraffin‐embedded (FFPE) myocardium were stained with H&E and consecutive sections with markers for different immune cell types using immunohistochemistry. Sections were stained for T cells (CD3 DAKO, A0452, 1:100), B cells (CD20 Roche, 790‐2531, undiluted), macrophages (CD68 Novocastra, NCL‐CD68‐KP1, 1:1600) and plasma cells (CD138 Serotec, MCA681A647, 1:500) using the Ventana automatic slide staining system. Detection of enzymatic activity was performed using diaminobenzidine (DAB). Histological sections were analysed using semi‐quantitative analysis. Immune cell infiltration was manually scored and classified into four phenotypes per immune cell type, ranging from 0 to 4 (0 = no inflammation, complete absence of infiltrating cells, 1 = mild inflammation, 0‐5 immune cells present per field, 2 = moderate inflammation, >5 immune cells diffusely present per field, 3 = moderate/severe inflammation, clusters of immune cells present, 4 = severe inflammation, excessive amount of infiltrating immune cells and clusters). Each histological section was assessed by randomly scoring 5 high power fields (magnification 400×) throughout the tissue section, which were averaged as a mean score per section. The scoring and classifications were determined by a certified pathologist and two independent observers blinded to section origin.

### 2.5 Immunofluorescence immunohistochemistry (IF) for the detection of immunoglobulins

Cryosections (8 μm) of human myocardium were incubated with FITC‐labelled anti‐IgG1 (Sigma, F0767, 1:30), FITC‐labelled anti‐IgG3 (Sigma, F4641, 1:15) and FITC‐labelled anti‐complement component 3 (C3c) (DAKO, F0201, 1:10). Sections of diseased kidney tissue (8 μm) served as positive control. Negative control stainings were included in which an antibody without fluorescent label was used. Slides were incubated with Sudan black (0.1%, Sigma‐Aldrich, 4197‐25‐5) for 20 minutes to limit background lipofuscin fluorescence. To visualize the localization of the antibodies in the myocardium, images were taken using a Zeiss Axiovert 200M microscope.

### 2.6 Tissue lysates of myocardium

Cryopreserved myocardium was cut into 10 sections of 10 μm and collected in tubes containing microbeads. Tissue extraction buffer ([100 mM Tris [pH 7.4]; Roche, 10708976001], 150 mM NaCl [Sigma‐Aldrich, S7653], 1 mM EGTA [Sigma‐Aldrich, 03777], 1 mM EDTA [Sigma‐Aldrich, E4884], 1% Triton X‐100 [Sigma, T8787] and 0.5% sodium deoxycholate [Sigma, 30970] dissolved in Mili‐Q water) was added, and the tissue was homogenized for 3× 35 seconds using a bead shaker (BioSpec). Constant agitation was maintained by rotating the lysates for 2 hours at 4°C. The samples were then centrifuged for 20 minutes (15 682 *g* at 4°C). Next, the supernatant was collected, aliquoted and stored at −80°C.

### 2.7 Multiplex immunoassay

Levels of IgM and IgG subclasses (IgG1, IgG2, IgG3, IgG4) were measured in tissue lysates and fresh plasma samples using a Bio‐Plex Pro™ Human Isotyping immunoassay 6‐plex (Bio‐Rad, 171A3100M) according to manufacturer's instructions. Plasma and tissue lysate immunoglobulin levels were calculated using internal standards.

### 2.8 IgG immunoprecipitation, gel electrophoresis and Western blot

Immunoprecipitation (IP) of IgG was performed according to manufacturer's protocol (Bio‐Rad). In brief, protein G‐coated magnetic beads (SureBeads™ Protein G Magnetic Beads; Bio‐Rad, 161‐4023) were washed with PBS‐T (PBS pH 7.4 and 0.1% Tween 20; EMD Millipore, 9005‐64‐5) and incubated with 1 μg of goat anti‐human IgG antibody (EMD Millipore, AP112, 1:400) for 1 hour. IgG‐coupled beads were incubated o/n with 15 μg protein from tissue lysates diluted in PBS. Magnetic beads were washed with PBS and dissolved in 40 μL Laemmli Buffer and 1% Nu‐Page sample reducing agent (Invitrogen, NP0004) and incubated for 10 minutes at 70°C. The precipitate was collected and used for gel electrophoresis and Western blotting (WB). Total of 15 μg protein per sample was loaded on pre‐casted Bolt 4%‐12% Tris‐Plus Gels (Invitrogen, NW04120BOX) for 1 hour at 160 V in MOPS SDS running buffer (Invitrogen, NP0001‐02). Proteins were transferred to PVDF membranes (Millipore, IPVH00010) and incubated o/n with a primary antibody (mouse anti‐human IgG; Novus, IG226, 1:400) and 1 hour with a secondary HRPO polyclonal Rabbit antimouse IgG (Dako, P0260, 1:2000). For visualization, a chemiluminescent peroxidase substrate (Sigma, CPS1120) was used and images were quantified using Image Lab Software (Bio‐Rad, 5.1 V).

### 2.9 Flow cytometry

Cryopreserved peripheral blood‐derived mononuclear cells (PBMCs), derived from five age‐ and sex‐matched end‐stage IHD patients and five matched end‐stage DCM patients, were collected. Peripheral blood‐derived mononuclear cells were thawed and washed with RPMI (61870010, Gibco) supplemented with GlutaMax (room temperature) containing 25 nM HEPES, 1% penicillin/streptomycin and 2% foetal bovine serum (FBS; 10270‐106, Gibco). Peripheral blood‐derived mononuclear cells were filtered over a 40‐μm cell strainer (542040, Greiner Bio‐One). The single cell suspension was added to an antibody mixture containing different cell surface markers to identify B‐cell subtypes as described before.^32^ Cells were stained with a fixable viability dye (eBioscience, eFluor‐506, 65‐0866‐14). Viable CD19^+^CD3^−^ B lymphocytes were selected for further gating of C24^−^CD38^+^ plasmablasts and CD27^−^, IgG^+^, CD24^+^, and CD38^+^ transitional/regulatory B cells using gating strategy as described by Meeuwsen et al.^32^ All appropriate controls were included in the experiments, including isotype/subclass‐matched primary antibody of irrelevant specificity. After flow cytometry, data were analysed using Kaluza 1.5a software (Beckman Coulter).

### 2.10 Statistical analysis

Statistical analysis and data representation were performed using IBM SPSS statistics 21 and GraphPad Prism© (GraphPad Software Inc version 7.02). Normal data distribution was tested, and normally distributed data were analysed using an unpaired *t* test. Non‐normally distributed data were compared using a Mann‐Whitney test. Group comparison was performed by a one‐way ANOVA or Kruskal‐Wallis test, corrected for multiple comparison testing. An UNIANOVA was used with age as covariate for the immunoglobulin analyses of the HELPFul cohort. Data are presented as mean ± SEM, unless stated otherwise. Values of *P* < .05 were considered significant.

## 3 RESULTS

### 3.1 High levels of IgG deposits in the myocardium of IHD patients with end‐stage HF

**Figure 1:**
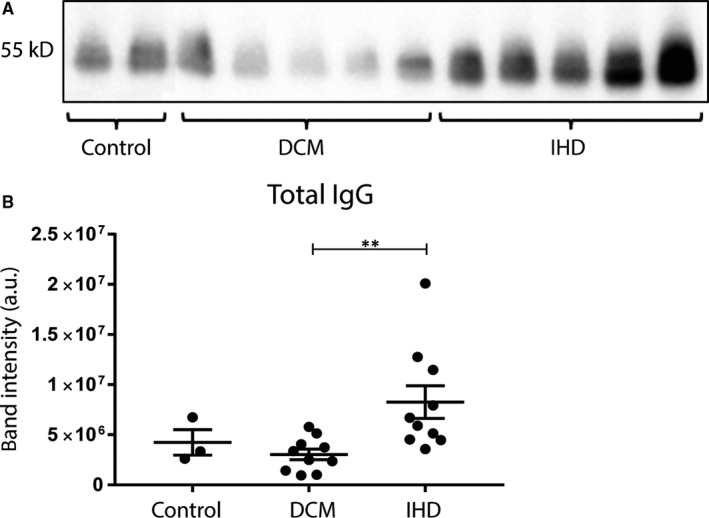
Deposits of IgG in myocardial lysates. Total IgG levels in myocardial lysates were determined using IP and subsequent WB (A). Quantification of band intensity is shown in (B), where total IgG levels were increased in IHD patients compared with DCM patients. DCM, dilated cardiomyopathy; IHD, ischaemic heart disease; IP, immunoprecipitation; WB, Western blot. Myocardial lysates: control n = 3, DCM n = 10, IHD n = 10. Significance was determined using one‐way ANOVA, ** *P* < .01

To investigate the presence of IgG deposits in the HFrEF patient cohort, myocardial lysates of the LV were used for immunoprecipitation. IgG precipitation followed by WB analysis showed high levels of IgG in myocardial lysates of IHD patients as compared to controls and DCM patients (Figure 1A). On average, myocardial IHD IgG levels were 2.7‐fold higher compared to DCM (*P* = .01) and 1.9‐fold higher compared to controls (not significant; Figure 1B).

### 3.2 Increased myocardial IgG3 and C3c deposits in end‐stage HF

**Figure 2:**
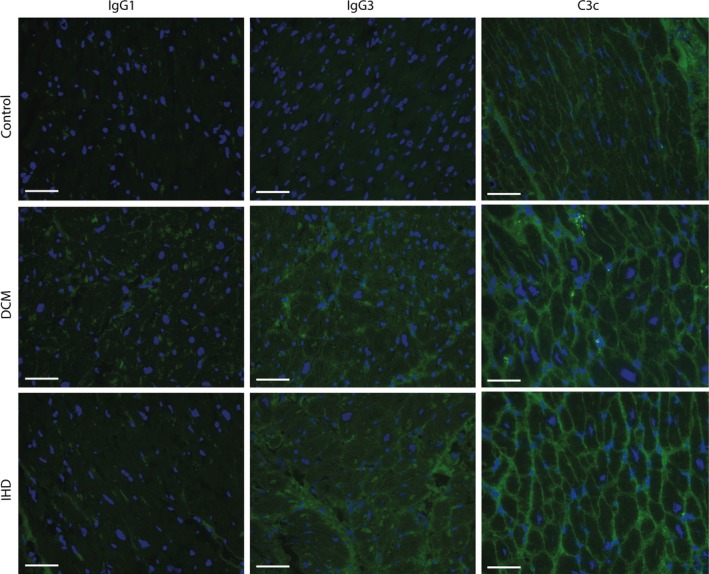
Deposition of IgG1, IgG3 and C3c in healthy and diseased myocardium. Cardiac tissue was fluorescently stained for IgG1, IgG3 and C3c (reflecting complement activation). DCM and IHD patients showed more deposits of IgG3 and C3c compared with control myocardium. IgG3 and C3c form an extensive network throughout the myocardium. Line bar indicates 50 µm, magnification 20×, C3c, complement factor 3c; DCM, dilated cardiomyopathy; IHD, ischaemic heart disease

To visualize the localization of IgG deposits in the myocardium and to investigate IgG subclasses and potential co‐binding with complement factor C3c, myocardial sections were fluorescently stained for IgG1, IgG3 and C3c (Figure 2). IgG1 showed no clear staining in both control and IHD/DCM patients. IgG3 was visible in IHD and DCM patients, but barely in controls. C3c was clearly elevated in end‐stage HF myocardium in both DCM and IHD patients, as compared to control myocardium. These findings indicate that IgG3 and C3c form a network of deposit throughout the myocardium of HF patients.

### 3.3 Increased numbers of myocardial immune cells in end‐stage HF

**Figure 3:**
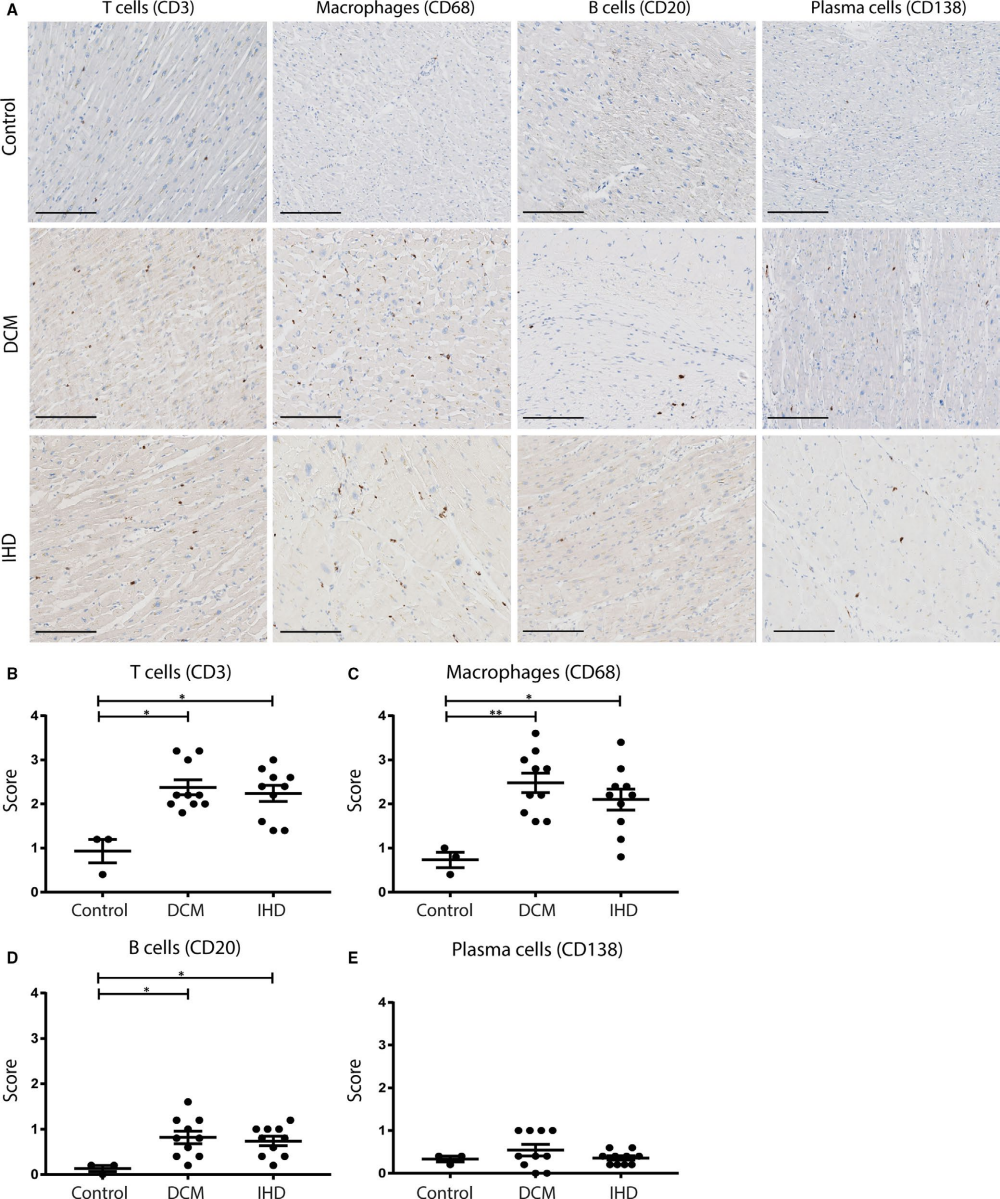
Infiltration of different immune cell types in the myocardium upon HF. Staining for T cells (CD3), macrophages (CD68), B cells (CD20) and plasma cells (CD138) in the myocardium of controls, DCM patients and IHD patients (A). The number of infiltrating immune cells was scored by semi‐quantitative analysis. End‐stage HF patients, both DCM and IHD, showed increased numbers of T cells (B), macrophages (C) and B cells (D). The number of plasma cells did not differ between the patient groups (E). Line bar indicates 200 µm, magnification 10×. DCM, dilated cardiomyopathy; IHD, ischaemic heart disease; HF, heart failure. Control n = 3, DCM n = 10, IHD n = 10. Significance was determined using one‐way ANOVA or Kruskal‐Wallis test, *P < .05, **P < .01

Next, we explored whether immunoglobulin deposits are accompanied with increased numbers of immune cells in the myocardium, ie T cells (CD3), macrophages (CD68), B cells (CD20) and plasma cells (CD138; Figure 3). Although traditionally thought to be linked only to acute MI, more infiltrating immune cells were observed in both the myocardium of DCM and IHD patients as compared to controls (Figure 3A). Semi‐quantitative analysis (Figure 3B‐D) showed a significantly higher number of CD3+ T cells, CD68+ macrophages and CD20+ B cells in both IHD and DCM patients, as compared to controls (T cells: DCM *P* = .02, IHD *P* = .02; macrophages: DCM *P* = .002, IHD *P* = .014; and B cells: DCM *P* = .02, IHD *P* = .04). Only a few CD138+ plasma cells were observed in the myocardium, and no difference in plasma cell numbers was observed between HF patients and controls (Figure 3E).

### 3.4 Increased IgG1 and IgG3 levels and decreased circulating regulatory B cells in HF patients

**Figure 4:**
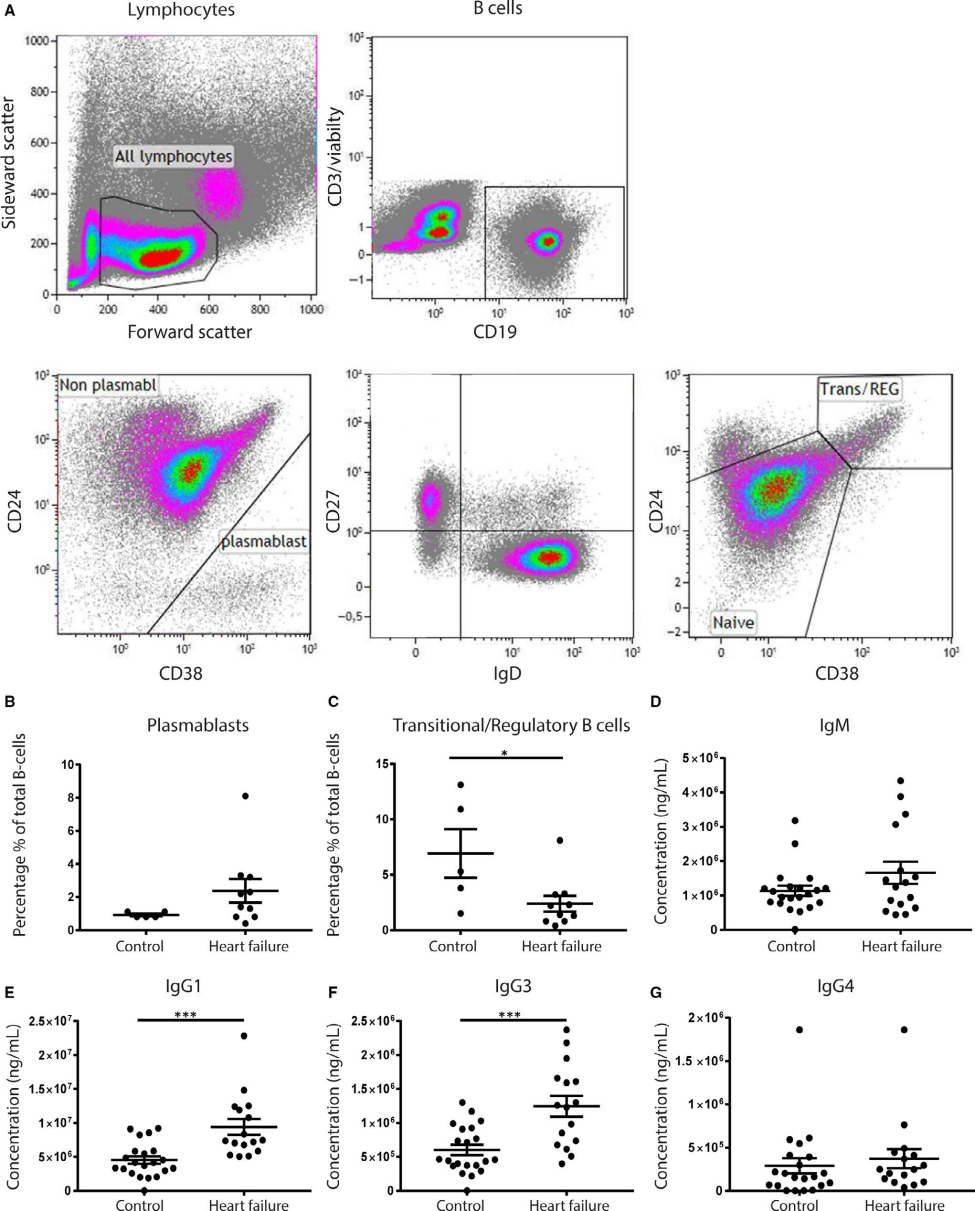
B‐cell subsets and immunoglobulin levels in HF patients. Flow cytometry on cryopreserved PBMC was used to identify different B‐cell subsets. (A) Gating strategy for selecting different B‐cell subsets. HF patients showed an increase in the percentage of plasmablasts (CD24‐CD38+), albeit not significant (B). Transitional/regulatory B cells, defined as CD38+CD24+, were decreased upon HF as compared to healthy controls (C). IgG1 and IgG3 levels were increased in HF patients (E‐F). IgM and IgG4 levels did not differ between the groups (D‐G). HF, heart failure; PBMC, peripheral blood mononuclear cells. For PBMCs: control n = 5, HF n = 10. For plasma samples: control n = 21, HF n = 16. Significance was determined using an unpaired *t* test. *P < .05, ***P < .001

Flow cytometry was performed on PBMCs derived from 10 end‐stage HFrEF patients (5 DCM and 5 IHD) and stained for different B‐cell markers to identify B‐cell subsets^32^ (Figure 4A). Data are presented as the percentage of total B‐cell count. The percentage of plasmablasts (CD24‐CD38+) in HF patients did not differ significantly from controls (*P* = .12; Figure 4B). The percentage of anti‐inflammatory transitional/regulatory B cells (CD27^−^, IgG^+^, CD24^+^, CD38^+^) was decreased in HF patients as compared to controls (*P* = .03; Figure 4C). Next, levels of IgM, IgG1, IgG3 and IgG4 were measured in freshly collected plasma samples of HF patients (n = 16) and compared to healthy controls (n = 21; Figure 4D‐G). IgG1 and IgG3 levels were significantly increased in patients with end‐stage HF compared with healthy controls (IgG1 *P* = .0003, IgG3 *P* = .0003; Figure 4E,F). IgM and IgG4 levels did not differ significantly between healthy controls and HF patients (IgM *P* = .11, IgG4 *P* = .56; Figure 4D‐G).

### 3.5 Circulating pro‐inflammatory markers mostly pronounced in IHD patients

**Figure 5:**
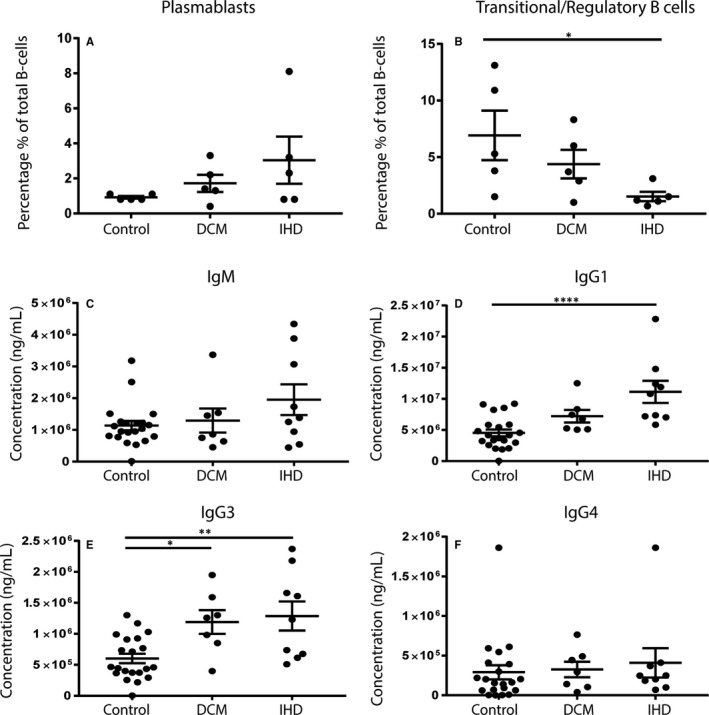
B‐cell subsets and immunoglobulin levels in IHD and DCM patients. When HF patients were divided into subgroups of DCM and IHD aetiology, IHD patients showed no significant increase in the percentage of plasmablasts (A) but did have a significantly lower number of transitional/regulatory B cells (B). There were no differences in IgM and IgG4 levels (C‐F). However, IgG1 and IgG3 levels were both significantly increased in IHD patients compared with healthy controls (D‐E). DCM, dilated cardiomyopathy; IHD, ischaemic heart disease; HF, heart failure. For PBMCs: control n = 5, DCM n = 5, IHD n = 5. For plasma samples: control n = 21, DCM n = 7, IHD n = 9. Significance was determined using one‐way ANOVA or Kruskal‐Wallis test, *P < .05, **P < .01, ****P < .0001

When the complete HF cohort was divided into DCM and IHD, the increase in percentage of plasmablasts was most pronounced in IHD. Furthermore, IHD patients showed significantly fewer transitional/regulatory B cells (CD38+CD24+; *P* = .04) as compared to healthy controls (Figure 5A,B). In addition, IgG1 and IgG3 levels were significantly increased in IHD patients (IgG1 = 1.1 × 10^7^ vs 4.5 × 10^6^ ng/mL, *P* < .0001, IgG3 = 1.3 × 10^6^ vs 6.0 × 10^5^ ng/mL, *P* = .002; Figure 5D‐E). IgG3 levels were also increased in DCM patients compared with healthy controls (IgG3 = 1.1 × 10^6^ vs 6.0 × 10^5^ ng/mL, *P* = .02). IgG1 levels showed a trend towards an increase, but did not reach statistical significance (IgG1 *P* = .09). IgM and IgG4 levels did not differ significantly between the groups (Figure 5C,F).

### 3.6 IgG1 and IgG3 levels as possible early markers of diastolic dysfunction in men

**Figure 6:**
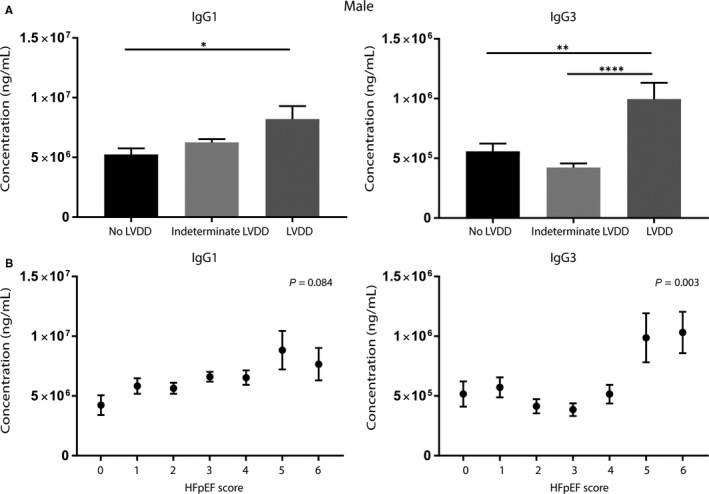
IgG1 and IgG3 in male patients with diastolic dysfunction. IgG1 and IgG3 levels measured in men with different stages of LVDD and levels (corrected for age). Men with LVDD showed significantly increased levels of IgG1 and IgG3 in plasma compared with those without LVDD (A). Increase in the HFpEF likelihood scores in men showed a significant trend towards higher IgG1 levels. Men with a HFpEF likelihood score of 5‐6 had significantly higher levels of IgG3 as compared to lower HFpEF likelihood scores (B). HF, heart failure; LVDD, left ventricular diastolic dysfunction. No LVDD (score 0‐1) n = 20, indeterminate LVDD (score 2‐4) n = 64 and LVDD (score 5‐6) n = 5. Significance was determined using an UNIANOVA, *P < .05, **P < .01 and ****P < .0001

To assess whether IgGs are possible markers for pre‐stage HF, we measured IgG1 and IgG3 levels in a cohort of patients, ranging from only slightly elevated filling pressures to more severe LVDD (Figure 6). Another advantage of this unique cohort is that it was specifically designed to assess sex differences in the progression of LVDD to HFpEF. Interestingly, men with LVDD show significantly increased levels of IgG1 (8.2 × 10^6^ vs 5.2 × 10^6^ ng/mL, *P* = .05) and IgG3 (1.0 × 10^6^ vs 5.5 × 10^5^ ng/mL, *P* < .0001) in the circulation as compared to men without LVDD (Figure 6A), when corrected for age. In addition, men with increasing HFpEF likelihood score showed an increasing trend in IgG1 levels (*P* = .084) and a significant increase in IgG3 (*P* = .003; Figure 6B). We did not find a correlation between IgG1 levels and C‐reactive protein (CRP) or brain natriuretic peptide (BNP) levels, confirming that these increased antibody levels are not part of a general inflammatory reaction (Tables S3 and S4). Strikingly, women with LVDD or increasing HFpEF likelihood score showed no difference in IgG1 and IgG3 levels as compared to women without LVDD (Figure S1), suggesting a male‐specific effect of the immunoglobulin levels in diastolic function. The levels of IgG4 and IgM did not differ between the different groups or men and women (data not shown).

## 4 DISCUSSION

The inflammatory response, in particular of T and B lymphocytes, plays an important role in adverse cardiac remodelling in HF.^28^ Furthermore, deposits of IgG can be found in end‐stage HFrEF myocardium,^27^, ^33^, ^34^ thereby suggesting the potential role of an (auto)‐immune response. However, the role of an antibody‐mediated immune response in HF progression is less clear and might differ between HF aetiologies. To investigate whether immune cell infiltration and immunoglobulin levels are associated with HF type and disease stage, we studied the immune response in patient cohorts with LVDD and late‐stage HFrEF, in which we also took sex differences into account.

In line with previous studies, we found an increased amount of IgG deposits in the myocardium of end‐stage HFrEF patients.^25^ This increase was significant in IHD patients as compared to DCM. Upon MI, secreted immunoglobulins are able to recognize cardiac‐specific antigens, which are suddenly exposed in great amounts upon ischaemia‐reperfusion injury. In this way, the immune system possibly becomes sensitized for these cardiac‐specific antigens. In DCM, the myocardial damage is less massive and therefore the immune system might be exposed to less cardiac‐specific epitopes possibly resulting in a less severe antibody‐mediated immune response. Binding of immunoglobulins to cardiac‐specific antigens on cardiomyocytes can initiate the process of complement activation and antibody‐dependent cellular cytotoxicity (ADCC), resulting in lysis of cardiomyocytes.^29^, ^35^, ^36^, ^37^ In DCM, it has also been described that immunoglobulins can directly bind to antigens expressed by the cardiomyocytes, and then crosslink to the Fc gamma receptor IIa on the cardiomyocyte, resulting in reduced calcium transients, cell shortening and initiating cellular apoptosis.^37^, ^38^, ^39^ The direct binding of immunoglobulins can also lead to complement‐dependent cytotoxicity (CDC). Using immunofluorescence, we observed an increased deposition of both IgG3 and C3c in the myocardial tissue of DCM and IHD patients. IgG3 has a high capability to activate components of the complement system,^40^ which probably explains the deposits of C3c in the failing hearts. Which specific epitopes are leading to this cascade is still under debate and is currently being investigated.

We also observed increased numbers of macrophages, T cells and B cells in the myocardium of HF patients compared with control myocardium. Long‐lived plasma cells migrate to the bone marrow, and this perhaps explains why we did not find increased numbers of plasma cells in the myocardium.^41^ The increased numbers of different inflammatory cell types in the chronically failing heart suggest a persistent low‐grade inflammatory response in these patients, which until now has mainly been described for the acute phases post‐MI and in myocarditis patients.^42^, ^43^

Despite low numbers of plasma cells in the myocardium of HF patients, we did find an increase in the number of plasmablasts in the circulation, albeit this fell short of being significant. Plasmablasts develop upon antigen‐stimulation and secrete immunoglobulins.^44^ The percentage of transitional/ regulatory B cells was decreased in IHD patients compared with healthy controls. This population is characterized by its immunosuppressive capacity, often mediated by IL‐10.^45^ A potential increase in the numbers of plasmablasts and a parallel/concomitant decrease in the numbers of immunosuppressive transitional/regulatory B cells, as observed in our IHD patients, might promote the putative autoimmune response in HF.

Consistent with our observations in the myocardium, we observed increased levels of IgG1 and IgG3 in fresh plasma samples of patients with end‐stage HF, most pronounced in IHD patients. Antibody responses to protein antigens are initiated via B cells and lead to the production of IgG1 and IgG3 specifically.^46^ Therefore, increased levels of IgG1 and IgG3 in end‐stage HF patients support the hypothesis of an immune response against cardiac proteins upon HF. Moreover, we showed that elevated levels of IgG1 and IgG3 are not limited to the end‐stage HFrEF population only, but can also already be found in patients with LVDD, the potential early phase of HFpEF. We demonstrated a significant correlation between the severity of LVDD and circulating levels of IgG1 and IgG3 in men. Possibly already in this early stage, small myocardial damage has occurred that sensitizes the immune system, for example due to microvascular dysfunction or increased wall pressure. In patients with indeterminate LVDD, IgG1 levels were starting to increase, whereas IgG3 levels were increasing at a later stage. This might be the initial phase of myocardial damage, leading to the production of IgG1 primarily, which is later accompanied by IgG3. Interestingly, the levels of IgG1 and IgG3 did not differ in women with LVDD, despite the fact that in general women are more prone to develop autoimmune diseases as compared to men.^47^, ^48^ In addition, women are more prone to develop HFpEF, whereas men more often develop HFrEF.^31^ What is causing this sex difference in the progression of LVDD to HFpEF is still poorly understood. Our findings suggest a possible difference in immune responses in early HF stages between men and women, thereby probably affecting HF progression.

Possible limitations of this study are the low number of control hearts and the low number of plasma samples used for the end‐stage HFrEF patients. Generally, the number of HTx is very limited because of the lack of donor hearts. In case a donor heart is not used for transplantation, and a consent is available to donate the heart to science, the heart can be used as a control heart. Therefore, the availability of control hearts for research purposes is extremely low. In addition, as only fresh plasma samples were collected upon HTx during the study, the number of plasma samples was limited to the numbers of HTx performed during the study. Another limitation is the low number of HFpEF patients in our cohort. HFpEF is a disorder with nonspecific HF‐like symptoms, which makes the diagnosis of the disease challenging. Diagnosis guidelines provided by the ESC are evolving over time, which leads to variation in the different classifications of HFpEF, and often also LVDD, upon each published guideline consensus paper. Nevertheless, the strong association of the immunoglobulins with the different groups of diastolic function in this heterogeneous population of patients at risk for LVDD underscores their potential pathogenic importance.

In conclusion, our study demonstrates an increased inflammatory status in end‐stage HFrEF, mostly pronounced in IHD, which includes increased amounts of IgG deposits, increased numbers of macrophages and lymphocytes in the myocardium, fewer transitional/regulatory B cells and increased levels of circulating IgG1 and IgG3. In addition, increased IgG1 and IgG3 levels already occur in patients with more severe LVDD, possibly before the development of clinical symptoms and signs, that is HFpEF, as shown in men with definite diastolic dysfunction. These findings support a role of ongoing sex‐dependent (auto)immune responses, starting in an pre‐clinical phase of LVDD, which has been shown to confer a higher risk of eventually developing HF. Therefore, this chronic immune response most likely influences progression of the disease. Our data suggest that increased levels of IgG1 and IgG3 may be useful biomarkers for early detection of HF progression, before clinical symptoms are present, which contribute to lifestyle and/or therapeutic intervention in an early stage of disease. Future studies should focus on the identification and validation of the epitopes recognized by autoantibodies. In addition, the role of IgG1 and IgG3 as potential markers for early HF recognition, screening and progression should be explored.

## CONFLICT OF INTEREST

None declared.

## AUTHOR CONTRIBUTION

PvdH, SdJ and JS wrote the manuscript. PvdH, SdJ, MH, RdW, AV and JS conceived and designed the study. PvdH, AS, YP, GV, MO and HdR acquired and analysed the data. PvdH, SdJ, MH, AS, GV, MO, RdW, PD, HdR, JL, AV and JS critically revised the manuscript for important intellectual content.

## Data Availability

Data are available upon request because of privacy/ethical restrictions.
