# Supplementary material for: Increased circulating IgG levels, myocardial immune cells and IgG deposits support a role for an immune response in pre‐ and end‐stage heart failure
Source: J Cell Mol Med. 2019 Sep 26;23(11):7505–16. doi: 10.1111/jcmm.14619 (PMC6815814; doi:10.1111/jcmm.14619)
Supplement: Supplementary file 1 [file JCMM-23-7505-s001.docx]

**Supplemental Baseline table 1**

| **Clinical characteristics of end-stage HF patients at time of transplantation**  **Overall n=20,** mean ± SD | |
| --- | --- |
|  |  |
| Patient age (years) | 57 ± 11.6 |
| Sex (% men) | 12 (60) |
| (% women) | 8 (40) |
| **Primary cardiac diagnosis** |  |
| *Ischemic heart disease (IHD)* |  |
| Number | 10 |
| BMI | 26.2 ± 2.18 |
| BNP (pmol/L) | 122.5 ± 110.8 |
| CRP (mg/L) | 7.76 ± 7.62 |
| eGFRcys (ml/min/1.73 m^2^) | 53.7 ± 14.7 |
| EF (%) | 20.0 ± 5.27 |
| LVMi (g/m2) | 109.7 ± 19.5 |
|  |  |
| *Dilated cardiomyopathy (DCM)* |  |
| Number | 10 |
| BMI | 20.61 ± 3.56 |
| BNP (pmol/L) | 394.7 ± 311.5 |
| CRP (mg/L) | 9.71 ± 6.49 |
| eGFRcys (ml/min/1.73 m^2^) | 78.0 ± 35.4 |
| EF (%) | 17.2 ± 6.18 |
| LVMi (g/m2) | 132.5 ± 38.5 |

**Supplemental Baseline table 1.** Clinical characteristics of end-stage HFrEF patients at time of heart transplantation.
