# Supplementary material for: Increased circulating IgG levels, myocardial immune cells and IgG deposits support a role for an immune response in pre‐ and end‐stage heart failure
Source: J Cell Mol Med. 2019 Sep 26;23(11):7505–16. doi: 10.1111/jcmm.14619 (PMC6815814; doi:10.1111/jcmm.14619)
Supplement: Supplementary file 2 [file JCMM-23-7505-s002.docx]

**Supplemental Baseline table 2**

| **Clinical characteristics of left ventricular diastolic dysfunction patients**  **Overall n=260,** mean ± SD | | |
| --- | --- | --- |
|  | **Men** | **Women** |
| Number (%) | 89 (34.2) | 171 (65.8) |
| Patient age (years) | 62.9 ± 9.4 | 63.5 ± 10.0 |
| BMI | 25.0 ± 14.3 | 27.4 ± 4.99 |
| HsTnl (pg/ml) | 4.00 ± 19.0 | 2.50 ± 10.5 |
| BNP (pg/ml) | 19.3 ± 66.9 | 21.9 ± 52.5 |
| CRP (mg/L) | 1.47 ± 14.9 | 1.74 ± 4.8 |
| eGFRcys (ml/min/1.73 m^2^) | 80.1 ± 12.0 | 76.8 ± 14.9 |
| EF (%) | 67.7 ± 8.57 | 66.8 ± 8.99 |
| LVMi (g/m2) | 80.2 ± 19.3 | 74.0 ± 18.5 |
| E/e’ ratio | 8.80 ± 2.79 | 9.49 ± 2.99 |
| E’ septal (cm/s) | 0.07 ± 0.02 | 0.07 ± 0.02 |
| E’ lateral (cm/s) | 0.09 ± 0.02 | 0.08 ± 0.02 |
| RWT | 0.42 ± 0.09 | 0.42 ± 0.10 |
| LAVi (ml/m2) | 24.2 ± 9.98 | 26.4 ± 13.3 |
| **Primary cardiac diagnosis** | | |
| ***No LVDD***  *HFpEF likelihood score 0-1* | **Men** | **Women** |
| Number | 20 | 27 |
| Patient age (years) | 57.2 ± 6.8 | 57.3 ± 7.3 |
| BMI | 27.0 ± 3.09 | 27.0 ± 4.24 |
| HsTnl (pg/ml) | 3.60 ± 26.3 | 2.10 ± 3.5 |
| BNP (pg/ml) | 12.8 ± 30.1 | 17.2 ± 13.6 |
| CRP (mg/L) | 1.57 ±16.7 | 3.10 ± 4.81 |
| eGFRcys (ml/min/1.73 m^2^) | 84.3 ± 14 | 80.0 ± 13.4 |
| EF (%) | 68.9 ± 7.99 | 65.5 ± 8.37 |
| LVMi (g/m2) | 77.5 ± 17 | 61.8 ± 19 |
| E/e’ ratio | 6.70 ± 2.02 | 8.00 ± 1.39 |
| E’ septal (cm/s) | 0.08 ± 0.01 | 0.09 ± 0.01 |
| E’ lateral (cm/s) | 0.11 ± 0.01 | 0.11 ± 0.02 |
| RWT | 0.38 ± 0.08 | 0.37 ± 0.06 |
| LAVi (ml/m2) | 22.7 ± 4.55 | 21.5 ± 5.70 |

| ***Indeterminate for LVDD***  *HFpEF likelihood score* *2-4* | **Men** | **Women** |
| --- | --- | --- |
| Number | 64 | 124 |
| Patient age (years) | 63.4 ± 9.5 | 63.1 ± 8.50 |
| BMI | 24.9 ± 16.5 | 27.6 ± 5.31 |
| HsTnl (pg/ml) | 3.90 ± 16.5 | 2.50 ± 10.1 |
| BNP (pg/ml) | 20.3 ± 27.8 | 21.3 ± 22.5 |
| CRP (mg/L) | 1.47 ± 11.0 | 1.65 ± 4.81 |
| eGFRcys (ml/min/1.73 m^2^) | 79.9 ± 10.7 | 77.0 ± 14.4 |
| EF (%) | 67.7 ± 7.82 | 67.2 ± 7.58 |
| LVMi (g/m2) | 79.1 ± 19.2 | 74.5 ± 15.0 |
| E/e’ ratio | 9.10 ± 1.89 | 9.75 ± 2.56 |
| E’ septal (cm/s) | 0.07 ± 0.02 | 0.06 ± 0.02 |
| E’ lateral (cm/s) | 0.08 ± 0.02 | 0.08 ± 0.02 |
| RWT | 0.44 ± 0.10 | 0.42 ± 0.10 |
| LAVi (ml/m2) | 23.7 ± 7.04 | 25.3 ± 8.53 |
| ***LVDD***  *HFpEF likelihood score* *5-6* | **Men** | **Women** |
| Number | 5 | 20 |
| Patient age (years) | 79.8 ± 5.4 | 73.9 ± 8.8 |
| BMI | 19.0 ± 10.6 | 26.2 ± 3.72 |
| HsTnl (pg/ml) | 15.5 ± 9.5 | 3.30 ± 17.2 |
| BNP (pg/ml) | 161 ± 185 | 82.3 ± 117 |
| CRP (mg/L) | 1.41 ± 37.2 | 1.36 ± 4.23 |
| eGFRcys (ml/min/1.73 m^2^) | 65.1 ± 9.17 | 72.0 ± 16.9 |
| EF (%) | 60.1 ± 16.4 | 66.1 ± 14.9 |
| LVMi (g/m2) | 104.1 ± 12.8 | 87.9 ± 27.8 |
| E/e’ ratio | 11.10 ± 6.44 | 12.4 ± 4.36 |
| E’ septal (cm/s) | 0.06 ± 0.01 | 0.05 ± 0.01 |
| E’ lateral (cm/s) | 0.09 ± 0.02 | 0.07 ± 0.02 |
| RWT | 0.42 ± 0.06 | 0.49 ± 0.08 |
| LAVi (ml/m2) | 51.0 ± 16.4 | 36.8 ± 25.96 |

**Supplemental Baseline table 2.** Clinical characteristics of patients with left ventricular diastolic dysfunction.
