# Supplementary material for: Increased circulating IgG levels, myocardial immune cells and IgG deposits support a role for an immune response in pre‐ and end‐stage heart failure
Source: J Cell Mol Med. 2019 Sep 26;23(11):7505–16. doi: 10.1111/jcmm.14619 (PMC6815814; doi:10.1111/jcmm.14619)
Supplement: Supplementary file 3 [file JCMM-23-7505-s003.docx]

**Supplemental table 3: IgG1 and IgG3 correlation with BNP-level in men**

| **Coefficients^a,b^** | | | | | | |
| --- | --- | --- | --- | --- | --- | --- |
| Model | | Unstandardized Coefficients | | Standardized Coefficients | t | Sig. |
|  |  | B | Std. Error | Beta |  |  |
| 1 | (Constant) | -150,119 | 45,836 |  | -3,275 | 0,002 |
|  | Age | 2,528 | 0,663 | 0,376 | 3,811 | 0,000 |
|  | IgG1ng/ml | 4,110E-06 | 0,000 | 0,138 | 1,393 | 0,167 |
| a. Gender_E1_C2 = men | | | | | | |
| b. Dependent Variable: BNP (pg/mL) | | | | | | |

| **Coefficients^a,b^** | | | | | | |
| --- | --- | --- | --- | --- | --- | --- |
| Model | | Unstandardized Coefficients | | Standardized Coefficients | t | Sig. |
|  |  | B | Std. Error | Beta |  |  |
| 1 | (Constant) | -148,641 | 39,785 |  | -3,736 | 0,000 |
|  | IgG3ng/ml | 8,028E-05 | 0,000 | 0,363 | 3,927 | 0,000 |
|  | Age | 2,286 | 0,621 | 0,340 | 3,682 | 0,000 |
| a. Gender_E1_C2 = men | | | | | | |
| b. Dependent Variable: BNP (pg/mL) | | | | | | |

**Supplemental table 3.** Correlation of IgG1 and IgG3 with BNP level in men with LVDD.
