# Supplementary material for: Increased circulating IgG levels, myocardial immune cells and IgG deposits support a role for an immune response in pre‐ and end‐stage heart failure
Source: J Cell Mol Med. 2019 Sep 26;23(11):7505–16. doi: 10.1111/jcmm.14619 (PMC6815814; doi:10.1111/jcmm.14619)
Supplement: Supplementary file 4 [file JCMM-23-7505-s004.docx]

**Supplemental table 4: IgG1 and IgG3 correlation with CRP-level in men**

| **Coefficients^a,b^** | | | | | | |
| --- | --- | --- | --- | --- | --- | --- |
| Model | | Unstandardized Coefficients | | Standardized Coefficients | t | Sig. |
|  |  | B | Std. Error | Beta |  |  |
| 1 | (Constant) | 1,946 | 11,038 |  | ,176 | ,860 |
|  | Age | -,021 | ,160 | -,014 | -,130 | ,897 |
|  | IgG1ng/ml | 8,757E-7 | 0,000 | ,132 | 1,232 | ,221 |
| a. Gender_E1_C2 = men | | | | | | |
| b. Dependent Variable: CRP (mg/L) | | | | | | |

| **Coefficients^a,b^** | | | | | | |
| --- | --- | --- | --- | --- | --- | --- |
| Model | | Unstandardized Coefficients | | Standardized Coefficients | t | Sig. |
|  |  | B | Std. Error | Beta |  |  |
| 1 | (Constant) | 2,676 | 9,845 |  | 0,272 | 0,786 |
|  | Age | -0,068 | 0,154 | -0,045 | -0,442 | 0,660 |
|  | IgG3ng/ml | 1,567E-05 | 0,000 | 0,319 | 3,098 | 0,003 |
| a. Gender_E1_C2 = men | | | | | | |
| b. Dependent Variable: CRP (mg/L) | | | | | | |

**Supplemental table 4.** Correlation of IgG1 and IgG3 with CRP level in men with LVDD.
