# Supplementary figures and images for: Increased circulating IgG levels, myocardial immune cells and IgG deposits support a role for an immune response in pre‐ and end‐stage heart failure
Source: J Cell Mol Med. 2019 Sep 26;23(11):7505–16. doi: 10.1111/jcmm.14619 (PMC6815814; doi:10.1111/jcmm.14619)

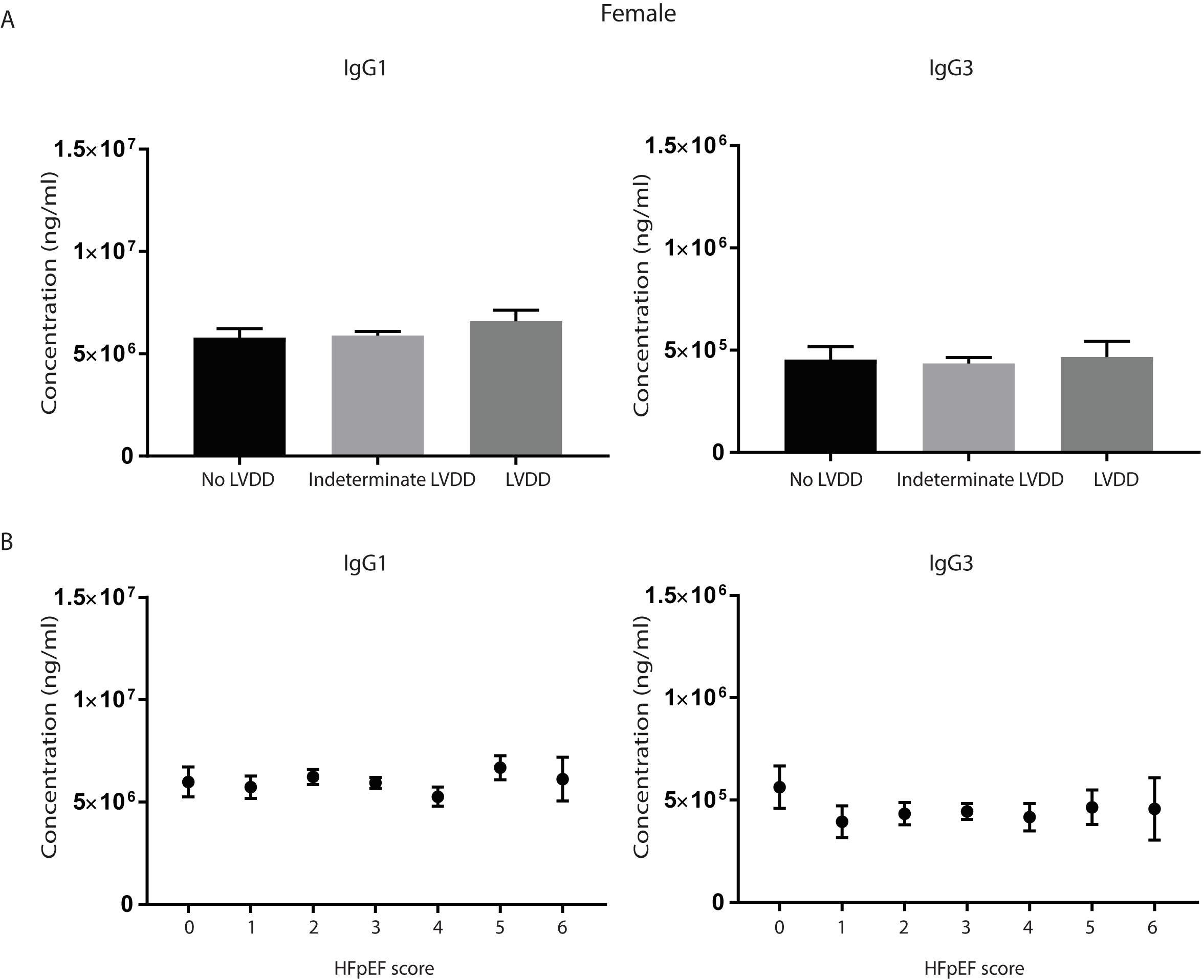

Supplement: Supplementary file 5 [file JCMM-23-7505-s005.tif]
